# Adjunctive use of enamel matrix derivatives to porcine‐derived xenograft for the treatment of one‐wall intrabony defects: Two‐year longitudinal results of a randomized controlled clinical trial

**DOI:** 10.1002/JPER.19-0432

**Published:** 2019-12-29

**Authors:** Jae‐Hong Lee, Do‐Hyung Kim, Seong‐Nyum Jeong

**Affiliations:** ^1^ Department of Periodontology, Daejeon Dental Hospital, Institute of Wonkwang Dental Research Wonkwang University College of Dentistry Daejeon Korea

**Keywords:** heterografts, periodontal diseases, randomized controlled trial, wound healing

## Abstract

**Background:**

The purpose of this study was to evaluate the potential advantages of adjunctive use of enamel matrix protein derivative (EMD) in combination with demineralized porcine bone matrix (DPBM) for the treatment of one‐wall intrabony defects in the molar regions, in comparison with the use of DPBM alone, through a randomized controlled clinical trial.

**Methods:**

Forty‐two participants were randomly assigned to two groups: one where DPBM with the adjunctive use of EMD (test group, n = 20) was applied and the other without EMD (control group, n = 22). Changes in the clinical and radiographic parameters from baseline at 6, 12, and 24 months were measured (probing pocket depth, clinical attachment loss, defect depth, and defect width). Postoperative discomfort (severity/duration of pain and swelling) and early soft tissue wound healing (dehiscence/fenestration, persistent swelling, spontaneous bleeding, and ulceration) were also assessed.

**Results:**

Both treatment modalities, with and without EMD, resulted in significant improvement of clinical and radiographic outcomes without any severe adverse events. However, no statistically significant differences in any of the measured parameters were found when the two groups were compared. Early wound healing outcomes and the severity of swelling did not differ between the groups, but the severity of pain (*P* = 0.046), duration (*P* = 0.033), and swelling (*P* = 0.022) were significantly lower in the test group.

**Conclusions:**

DPBM has been verified for biocompatibility and can be used as a scaffold to enhance the clinical and radiographic outcomes of periodontal regeneration of one‐wall intrabony defects. In particular, the adjunctive use of EMD significantly reduced the postoperative discomfort.

## 1 INTRODUCTION

The guided tissue regeneration (GTR) technique has been considered the gold standard for periodontal regenerative treatment for the past several decades.^1^, ^2^ However, alternate treatment modalities and materials have been introduced and applied recently to overcome the technique sensitivity and critical complications, and to avoid additional surgeries of the GTR technique.^3^, ^4^ Despite these steady efforts, achieving complete and functional periodontal regeneration with the re‐formation of a new periodontal ligament, new cementum with inserted principal collagen fibers, and new alveolar bone is still challenging.^5^

Enamel matrix protein derivative (EMD), derived from developing porcine teeth, induces the expression of cementogenesis‐ and osteogenesis‐related genes in the mesenchymal cells, and has been used as an osteo‐promotive agent for the regeneration of damaged periodontal support structures for >20 years.^6^, ^7^ In recent years, a number of studies of non‐surgical and surgical periodontal treatments with the adjunctive use of EMD that show improvements in clinical, radiographic, and histological parameters have been conducted and published.^8^, ^9^, ^10^ These findings are supported by the Cochrane database meta‐analyses, but the high degree of heterogeneity in the included clinical trials impedes a clear demonstration.^11^

Previous studies have evaluated the effect of combinations of EMD with bone grafts, including autogenous bone, allogenous bone, xenografts, and allografts, for the clinical and radiographic resolution of periodontal intrabony defects.^12^, ^13^ In particular, among the various grafting and scaffold materials, the good biocompatibility and osteoconductivity of demineralized bovine bone matrix, which is one of the most studied and accepted xenograft biomaterials, has been verified. It has been widely used to improve the results of regenerative treatment in combination with EMD.^13^, ^14^, ^15^

Demineralized porcine bone matrix (DPBM), with structure and composition similar to that of human bones, was developed more recently and has been successfully used in the field of dentistry.^16^, ^17^ Histologic and ultrastructural results from using DPBM for maxillary sinus augmentation surgery showed good biocompatibility and that DPBM did not interfere with the normal bone healing process.^18^ Similarly, alveolar ridge preservation with DPBM showed reduced buccal bone resorption and maintained volume stability in intact and damaged extraction sockets.^19^, ^20^ However, there have been few clinical studies that focused on the benefits of using DPBM for periodontal regeneration in cases of intrabony defects. To the best of our knowledge, there are no studies that directly compare the outcomes of DPBM with and without the adjunctive use of EMD for the treatment of one‐wall intrabony defects. Therefore, the purpose of this study is to evaluate the potential advantages of the adjunctive use of EMD in combination with DPBM for the treatment of one‐wall intrabony defects in the molar regions when compared with the use of DPBM alone, through a randomized controlled clinical trial.

## 2 MATERIALS AND METHODS

### 2.1 Ethical statements

**Figure 1:**
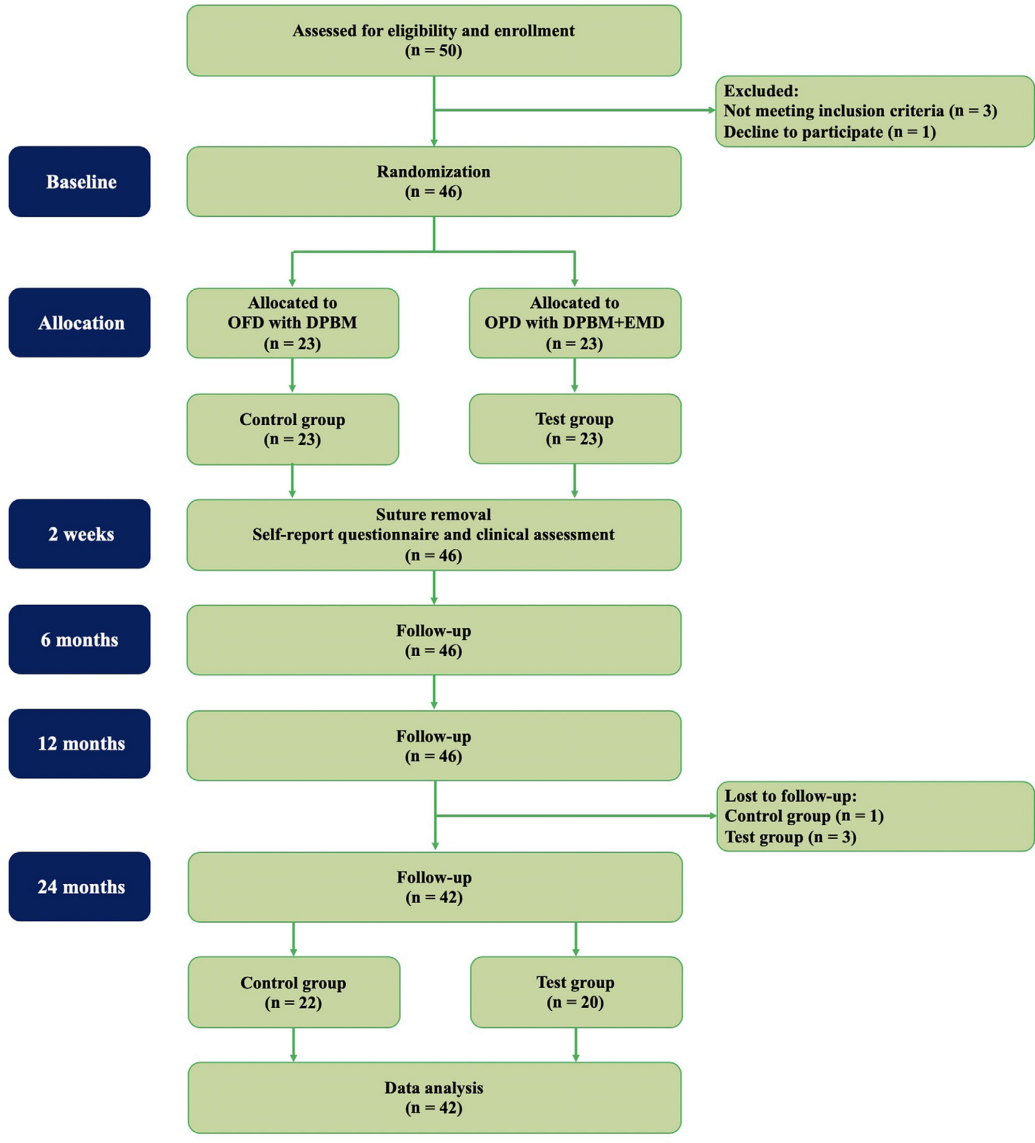
Flowchart of inclusion and exclusion criteria. The reporting of this prospective, randomized, controlled, clinical trial has followed the Consolidated Standards of Reporting Trial (CONSORT) guidelines. OFD, open flap debridement; EMD, enamel matrix derivative; DPBM, demineralized porcine bone matrix

All protocols and materials were approved by the Institutional Review Board (IRB) of Daejeon Dental Hospital, Wonkwang University (approval No. W1612/003‐002). All enrolled participants provided written, fully informed consent in accordance with the IRB guidelines, and the study was conducted in accordance with the Helsinki Declaration of 1975, as revised in 2013.^21^ The trial was registered with the Republic of Korea Clinical Trials Registry (Identifier Number: KCT0004164), and all procedures were performed between January 2017 and July 2017. The Consolidated Standards of Reporting Trial (CONSORT) flowchart for randomized controlled clinical trial is presented in Figure 1.^22^

### 2.2 Study design and population

This study is a prospective, randomized controlled clinical trial with a parallel design and 2‐year duration. A total of 50 participants who underwent open flap debridement (OFD) were recruited from the Department of Periodontology at Daejeon Dental Hospital, Wonkwang University, between January 2016 and December 2016. The following inclusion criteria were applied: 1) aged ≥19 years, 2) must have completed conventional periodontal treatment (scaling and root planing) at the surgical site, 3) presence of a one‐wall intrabony defect (>3 mm) in molar teeth, 4) healthy or well‐controlled systemic conditions for surgical intervention (patients were American Society of Anesthesiology [ASA]‐1 or ASA‐2), 5) no or well‐controlled diabetes, and 6) adequate plaque control and stable periodontal status (full‐mouth bleeding score on probing: <25%, and full‐mouth plaque score: <25%).^23^, ^24^ The following exclusion criteria were established: 1) intrabony defects extending into furcation area, 2) OFD completed within 6 months at the same surgical site, 3) pregnant or lactating women, 4) current smokers, and 5) those who did not fully understand the purpose of the experiment and did not provide informed consent.

### 2.3 Primary and secondary outcomes

- Primary outcomes: Changes in the clinical and radiographic parameters of one‐wall intrabony defects from baseline, at 6, 12, and 24 months after treatment. Clinical and radiographic parameters included probing depth (PD), clinical attachment level (CAL), defect depth, and defect width.
- Secondary outcomes: Postoperative discomfort (severity/duration of pain and swelling) and early soft tissue wound healing (spontaneous bleeding, persistent swelling, and ulceration) during the 2 weeks after OFD.

### 2.4 Randomization and study group allocation

The required sample size calculation was performed using software.1G*Power version 3.01 software, Franz Faul, Christian‐Albrechts‐University, Kiel, Germany. The sample size was based on previous studies (difference in CAL changes as the primary outcome parameter of 0.5 mm and standard deviation of 0.8 mm) and the significance level was set at 0.05 and power of 80%.^25^, ^26^ Fifty participants were included, and a 10% dropout rate was considered. Out of the 50 participants who underwent OFD, four were excluded for the following reasons: did not meet the inclusion criteria (n = 3) and declined to participate (n = 1). From the 46 remaining eligible participants, randomization and allocation were performed before the intervention by the independent allocator. Each participant was randomly assigned with a 1:1 allocation ratio to the control (n = 23) and test (n = 23) groups using computer‐generated random numbers, with random block sizes of two and four.

### 2.5 Surgical treatment

All OFD were conducted by one experienced periodontology specialist (JHL). Participants were administered an antibiotic (netilmicin 50 mg/2 mL) and analgesic (diclofenac 90 mg/2 mL) injection 30 minutes before OFD, and were provided with postoperative medication (antibiotics [amoxicillin 500 mg three times a day], analgesics [ibuprofen 200 mg three times a day]), and mouthwash2GUM Activital Dental Conditioner AN, Sunstar, Osaka, Japan. to be used for 5 days after OFD. Intrasulcular incision was performed, and a full‐thickness mucoperiosteal flap was elevated minimally by using #12, #15, and #15c blades and an Orban knife under appropriate local anesthesia (2% lidocaine HCl with 1:100,000 epinephrine) to expose the one‐wall intrabony defect. Granulation tissue was carefully and gently removed using curets3Standard and mini Gracey curets, Hu‐friedy, Chicago, IL. and an ultrasonic device.4SONICflex air scaler, KaVo, Biberach, Germany.


#### 2.5.1 Test group

Root conditioning and decontamination were performed using tetracycline at a concentration of 50 mg/mL; the tetracycline‐soaked cotton pellets were applied to the exposed root surfaces and intrabony defects for 2 minutes. EMD5Emdogain 0.3 mL, Straumann, Basel, Switzerland. was applied to the root surface, followed by appropriate filling of the mixture of DPBM6THE Graft 0.25 g, Purgo Biologics, Seongnam, Korea. and EMD to the level of the alveolar crest bone.

#### 2.5.2 Control group

The intrabony defects were filled with DPBM, without adjunctive EMD.

The flaps were repositioned, and tension‐free primary closure was achieved using modified horizontal mattress and interrupted sutures with a non‐absorbable polytetrafluoroethylene monofilament.7Biotex, Purgo Biologics, Seongnam, Korea. After 2 weeks, the sutures were removed, and the participants were instructed to perform soft tooth cleaning in the surgical site. Routine clinical and radiographic follow‐up with prophylaxis were scheduled at 6, 12, and 24 months.

### 2.6 Clinical and radiographic analysis

All clinical and radiographic outcomes were investigated by a single calibrated examiner (JHL) at 6, 12, and 24 months. To evaluate the intra‐examiner reliability, 10 cases were scored twice, and this showed acceptable levels (good to excellent) of agreement (>0.80).

#### 2.6.1 Clinical outcomes

PD was measured as the distance between the gingival margin and the most apical extension of the clinical gingival sulcus. Gingival recession was measured as the distance between the gingival margin and the cemento‐enamel junction, and CAL was measured as the sum of PD and gingival recession. Clinical outcomes were recorded to the nearest millimeter with a periodontal probe.8CP 15 UNC periodontal probe, Hu‐Friedy, Chicago, IL.


#### 2.6.2 Radiographic outcomes

Defect depth was measured as the distance between the alveolar bone crest and the most apical extension of the bone defect, and defect width was measured as the horizontal distance between the alveolar bone crest at the intrabony defect in the direction towards the center of the tooth. Radiographic outcomes were measured using the medical imaging viewer.9Osirix X 10.0 64‐bit version software, Pixmeo SARL, Geneva, Switzerland.


### 2.7 Patient‐reported outcomes and clinical assessment

All enrolled participants filled out the self‐report questionnaire or chose to have the questionnaire read out by a trained periodontist at the end of the suture removal visit (2 weeks after OFD). The subjective severity of pain and swelling at the surgical site was evaluated using a visual analog scale which presented: 0 (no pain and swelling) to 10 (worst imaginable pain and swelling). The duration of subjective pain and swelling were also measured during the 2 weeks after OFD. During suture removal, soft tissue wound healing outcomes (including dehiscence/fenestration, persistent swelling, spontaneous bleeding, and ulceration) at the surgical site were assessed by a single calibrated examiner (JHL).

### 2.8 Statistical analysis

Chi‐square tests and independent *t* tests were conducted to compare primary and secondary outcomes of the control and test groups. Frequencies (*n*), proportions (%), mean (mm), and standard deviation (mm) values for the ordinary and continuous variables, and the range of error at 95% confidence intervals (CIs) were computed for descriptive analyses of the data. All calculations were performed using statistical software,10SAS version 9.4 software, SAS Institute, Cary, NC. and a *P* value <0.05 was considered statistically significant.

## 3 RESULTS

### 3.1 Baseline characteristics

The 42 participants included, based on the inclusion and exclusion criteria, comprised 20 (47.6%) males and 22 (52.4%) females with a mean age of 54.6 ± 11.4 years. No statistically significant difference was observed in oral health‐related factors (full‐mouth bleeding on probing score and full‐mouth plaque score), operation time, and diabetes mellitus between the control and test groups (Table 1).

**Table 1:** Demographic and clinical parameters of participants who underwent open flap debridement with DPBM only (control group) and DPBM + EMD (test group)

	Control group (n = 22)		Test group (n = 20)		
Variables	Total no.	%	Total no.	%	*P*
Demographic factors					
Sex					
Male	12	54.5	8	40.0	0.526
Female	10	45.5	12	60.0	
Age (years)	55.9 ± 13.0		53.4 ± 9.5		0.479
Oral health‐related factors					
FMBS	28.9 ± 19.1		24.4±20.2		0.342
FMPS	29.2 ± 11.7		27.2±20.6		0.681
Operation time (minutes)	38.5 ± 11.1		41.3 ± 9.9		0.343
Comorbid disease					
Diabetes mellitus	5	22.7	6	30.0	0.868

*P* values were calculated using the Chi‐square and independent *t* test.

DPBM, porcine bone‐derived ceramic bone graft substitute; EMD, enamel matrix derivate; FMBS, full‐mouth bleeding on probing score; FMPS, full‐mouth plaque score.

### 3.2 Primary outcomes

**Figure 2:**
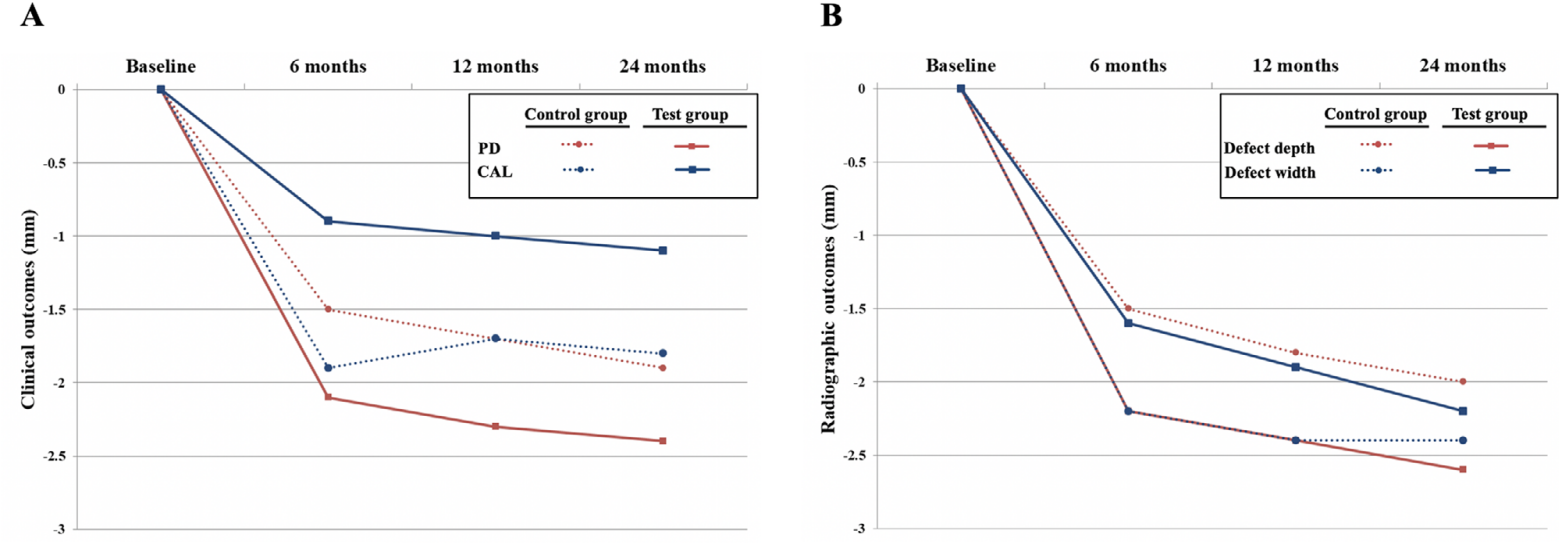
Comparison of clinical and radiographic outcomes between control and test groups after treatment for one‐wall intrabony defects: (**A**) Clinical outcomes at 6, 12, and 24 months measured as probing pocket depth and clinical attachment loss; (**B**) Radiographic outcomes at 6, 12, and 24 months measured as defect depth and width

The clinical and radiographic outcomes are presented in Table 2 and Figure 2. Clinically, at 6 months after the periodontal treatment, the control group showed a significant reduction in PD from 7.3 ± 0.6 mm to 5.8 ± 0.8 mm (*P* <0.001) and a significant change in CAL from 7.8 ± 0.6 mm to 7.0 ± 0.9 mm (*P* <0.001). Radiographically, also a significant change in defect depth from 4.3 ± 0.6 mm to 2.8 ± 0.4 mm (*P* <0.001), and a change in defect width from 3.3 ± 0.6 mm to 1.7 ± 0.4 mm (*P* <0.001) were observed. At 6 months, the test group showed a significant reduction in PD from 7.8 ± 1.0 mm to 5.7 ± 0.7 mm (*P* <0.001) and a significant change in CAL from 8.5 ± 1.3 mm to 7.0 ± 1.0 mm (*P* <0.001). Radiographically, also a significant change in defect depth from 4.6 ± 0.8 mm to 2.3 ± 0.9 mm (*P* < 0.001), and a change in defect width from 3.5 ± 0.9 mm to 1.3 ± 0.5 mm (*P* < 0.001) were also observed. However, after 12 months of periodontal treatment, all variables in the test group and clinical variables in the control group showed no significant difference between the 6‐month results except for radiographic outcomes in the control group. Furthermore, after 24 months, only the control group's radiographic defect depth showed a significant difference from the 12‐month results.

**Table 2:** Clinical and radiographic outcomes at baseline, 6, 12, and 24 months after treatment of one‐wall intrabony defects

	Control group							Test group						
	Baseline	6‐month follow‐up		12‐month follow‐up		24‐month follow‐up		Baseline	6‐month follow‐up		12‐month follow‐up		24‐month follow‐up	
Parameters (mm)	Mean ± SD (95% CI)	Mean ± SD	*P* ^1)^	Mean ± SD	*P* ^2)^	Mean ± SD	*P* ^3)^	Mean ± SD	Mean ± SD	*P* ^1)^	Mean ± SD	*P* ^2)^	Mean ± SD	*P* ^3)^
Clinical outcomes														
PD	7.3 ± 0.6	5.8 ± 0.8	**<0.001**	5.5 ± 0.7	0.208	5.4 ± 0.8	0.625	7.8 ± 1.0	5.7 ± 0.7	**<0.001**	5.4 ± 0.6	0.240	5.4 ± 0.7	0.835
	(7.0‒7.5)	(5.5‒6.1)		(5.2‒5.8)		(5.1‒5.7)		(7.3‒8.2)	(5.4‒6.0)		(5.2‒5.7)		(5.1‒5.7)	
CAL	7.8 ± 0.6	7.0 ± 0.9	**<0.001**	6.8 ± 0.8	0.489	6.7 ± 0.9	0.815	8.5 ± 1.3	7.0 ± 1.0	**<0.001**	7.0 ± 0.9	0.904	6.9 ± 0.8	0.768
	(7.6‒8.1)	(6.6‒7.3)		(6.4‒7.1)		(6.4‒7.1)		(7.9‒9.1)	(6.6‒7.4)		(6.6‒7.4)		(6.6‒7.3)	
Radiographic outcomes														
Defect depth	4.3 ± 0.6	2.8 ± 0.6	**<0.001**	2.5 ± 0.4	**0.020**	2.3 ± 0.5	0.302	4.6 ± 0.8	2.6 ± 0.8	**<0.001**	2.3 ± 0.9	0.146	2.1 ± 0.8	0.535
	(4.0‒4.5)	(2.6‒3.1)		(2.3‒2.6)		(2.1‒2.5)		(4.3‒5.0)	(2.3‒3.0)		(1.9‒2.6)		(1.7‒2.4)	
Defect width	3.3 ± 0.6	1.7 ± 0.4	**<0.001**	1.4 ± 0.3	**0.012**	1.1 ± 0.5	**0.030**	3.5 ± 0.9	1.3 ± 0.5	**<0.001**	1.1 ± 0.6	0.302	1.0 ± 0.5	0.723
	(3.0‒3.5)	(1.5‒1.9)		(1.2‒1.5)		(0.9‒1.3)		(3.1‒3.9)	(1.0‒1.5)		(0.8‒1.3)		(0.8‒1.2)	

Mean values ± SD are presented; boldface denotes statistical significance (*P* < 0.05).

CAL, clinical attachment level; PD, probing depth; .

*P* values for comparisons between 1) baseline versus 6 months, 2) 6 months versus 12 months, and 3) 12 months versus 24 months, respectively.

### 3.3 Secondary outcomes

**Figure 3:**
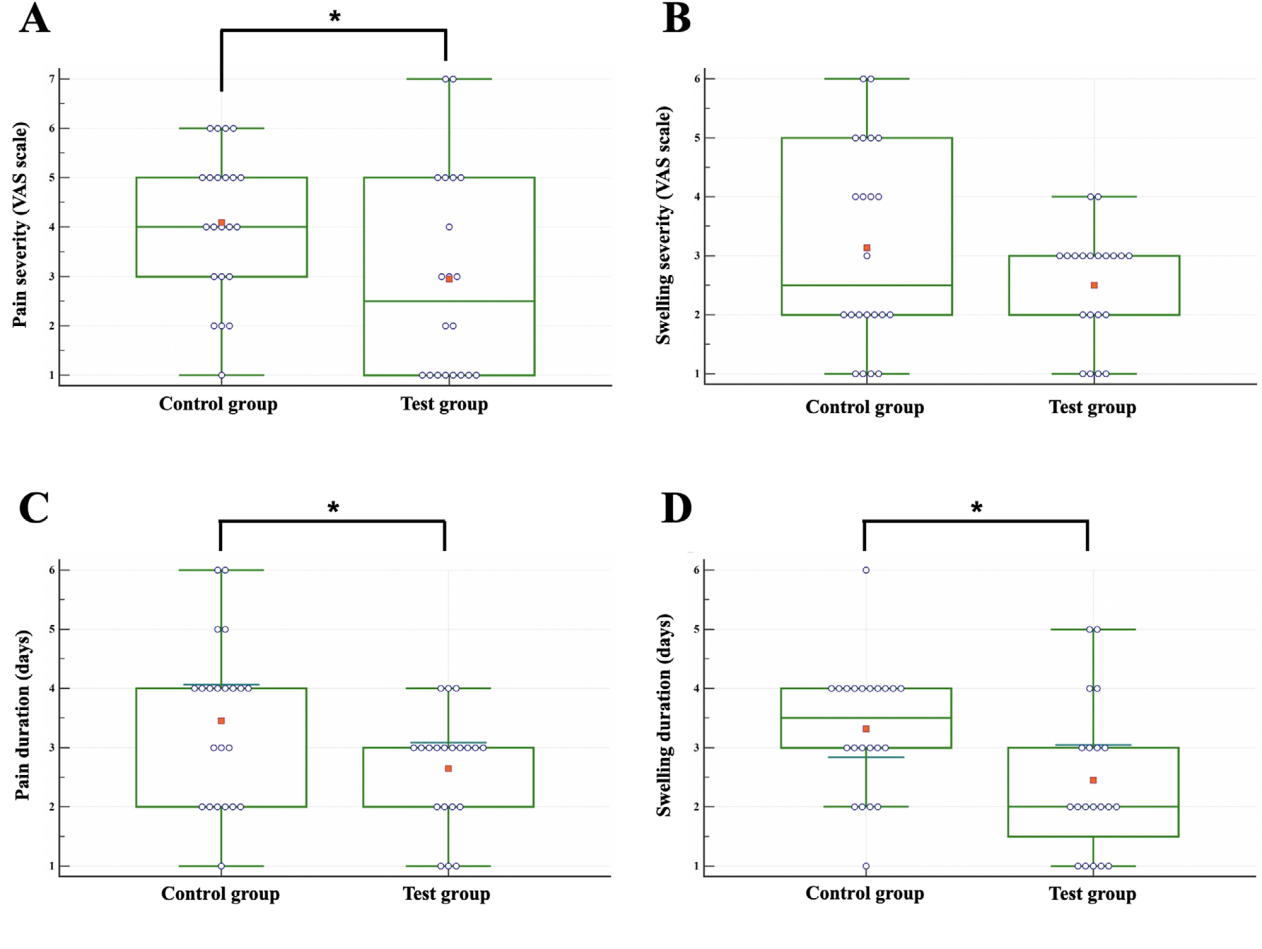
Comparison between control and test groups of early postoperative discomfort at the end of the suture removal visit (2 weeks after OFD). (**A** and **B**) show the severity of pain and swelling using visual analogue scale (VAS) and (**C** and **D**) show the durations of pain and swelling, respectively. Statistically significant differences between the two groups were determined (*P <0.05). Data are presented using box‐whisker plots (25th and 75th percentile [green box], mean [red dot], and outliers)

The severity of swelling (difference [Df] = 0.63, 95% CI = −0.23 to 1.50; *P* = 0.147) did not differ between the control and test groups, but the severity of pain (Df = 1.14, 95% CI = 0.02 to 2.26; *P* = 0.046) was significantly lower in the test group than the control group. The duration of pain (Df = 0.80, 95% CI = 0.06 to 1.54; *P* = 0.033) and swelling (Df = 0.86, 95% CI = 0.13 to 1.60; *P* = 0.022) were also significantly reduced in the test group (Fig. 3). In the control group, dehiscence and/or fenestration occurred in four patients (18.2%), persistent swelling occurred in six (27.3%), spontaneous bleeding occurred in two (9.1%), and ulceration occurred in one (4.5%). In the test group, the same complications occurred in one patient (5.0%), two (10.0%), one (5.0%), and two (10.0%), respectively. No statistically significant differences were observed between the control and test groups, with regard to any early clinical complications (Table 3).

**Table 3:** Clinical assessment of soft tissue wound healing outcomes during suture removal

	Control group (n = 22)		Test group (n = 20)		
Variables	Total no.	%	Total no.	%	*P*
Dehiscence and/or fenestration					
No	18	81.8	19	95.0	0.400
Yes	4	18.2	1	5.0	
Persistent swelling					
No	16	72.7	18	90.0	0.302
Yes	6	27.3	2	10.0	
Spontaneous bleeding					
No	20	90.9	19	95.0	0.931
Yes	2	9.1	1	5.0	
Ulceration					
No	21	95.5	18	90.0	0.931
Yes	1	4.5	2	10.0	

*P* values were calculated using the Chi‐square test.

## 4 DISCUSSION

The current study showed that both treatment modalities, DPBM with and without EMD, for the treatment of one‐wall intrabony defects in molar regions resulted in significant improvements of clinical and radiographic outcomes without any severe adverse events. However, there were no statistically significant differences in any of the measured parameters in comparison of the two groups. These results demonstrated that OFD with DPBM is an effective treatment method, and that DPBM used as a scaffold for the treatment of intrabony defects, exhibits good biocompatibility.

Space provision and maintenance are essential factors and more important than tissue occlusion in achieving periodontal regeneration in intrabony defects.^27^ Although EMD with semi‐fluid properties does not have space‐making and retention capabilities, several studies have revealed a clinical advantage in the adjunctive use of EMD for the treatment of intrabony defects, and treatment with adjunctive EMD was better than that of OFD alone.^25^, ^28^, ^29^ Human histometrical results also indicated that OFD with the adjunctive use of EMD may enhance the formation of new bone, cementum, and connective tissue attachment in intrabony defects.^30^, ^31^ In particular, considering the results of periodontal treatment performed on the three‐wall intrabony defects, OFD with EMD alone or OFD with EMD combined with other biomaterials (including barrier membrane and grafting material) showed more favorable clinical, radiographic, and histological outcomes when compared with the results from the use of OFD alone.^31^, ^32^

Adjunctive use of bone grafting material with EMD led to a statistically significant PD reduction and CAL gain, and promoted filling of the defect with newly formed bone when compared with the cases where OFD with EMD or with bone grafting alone was used for the treatment of intrabony defects.^14^, ^26^, ^33^ Despite these favorable outcomes, systematic reviews and meta‐analyses involving one‐, two‐, and three‐wall intrabony defects concluded that the treatment of intrabony defects with the adjunctive use of EMD in combination with bone grafting material did not result in clinically or radiographically superior results over that from the use of OFD with bone grafting.^32^, ^34^ Similarly, our current study showed an average CAL gain of 1.3 ± 1.2 mm and PD reduction of 2.1 ± 1.1 mm, and there was also no statistically significant difference between the control and test groups. The improvement of primary outcomes is lower than that of previous one‐wall defect studies, these results are considered to reflect the fact that the current study was limited to only one‐wall intrabony defects in the molar regions.

Amelogenin, a major component of EMD, plays the important role of promoting fibroblast adhesion and proliferation, consequently improving the biological activity in the early wound healing process, post‐operative morbidity, and patient‐centered subjective outcomes.^35^, ^36^ Our previous studies have shown that the severity of pain and swelling did not differ between the test (OFD/alveolar ridge preservation with EMD) and control (OFD/alveolar ridge preservation without EMD) groups; however, the duration of pain (*P* <0.05) and swelling (*P* <0.05) were significantly lower in the test group.^37^, ^38^ Similar to the previous studies, the severity of pain (Df = 1.14, 95% CI = 0.02 to 2.26; *P* = 0.046) and duration of pain (Df = 0.80, 95% CI = 0.06 to 1.54; *P* = 0.033) and swelling (Df = 0.86, 95% CI = 0.13 to 1.60; *P* = 0.022) were significantly lower in the test group, except for the severity of postoperative swelling (Df = 0.63, 95% CI = −0.23 to 1.50; *P* = 0.147).

On the contrary, no statistically significant differences with regard to any early clinical complication including dehiscence/fenestration, persistent swelling, spontaneous bleeding, and ulceration were observed between the control and test groups. Although these findings confirm the tendency of the adjunctive use of EMD to promote periodontal regeneration and reduce postoperative discomfort, it is difficult to definitively make this conclusion, given the data provided in the study. In particular, since OFD technique itself is minimally invasive and less gives soft tissue damage, it is considered as one of the major reasons why the adjunctive use of EMD is not significantly effective in soft tissue wound healing outcomes. Therefore, further studies need to confirm whether application of EMD promotes early wound healing in surgeries with extensive soft tissue damage.

The current study has several limitations. First, this study included both maxillary and mandibular molars with one‐wall intrabony defects. Various tooth positions and defect morphologies are mixed and confused, so careful interpretation is necessary to prevent heterogeneity. In addition, further research is also necessary to evaluate the efficacy of adjunctive use of EMD in combination with DPBM for the treatment of anterior and premolar one‐wall intrabony defects. Second, in our study, four of 46 participants (one in control and three in the test groups) were unable to complete all study‐related visits. Due to the relatively small sample size, our results should be interpreted conservatively. Third, participants may experience memory bias about the post‐operative discomfort. Since the degree of pain and swelling is highly subjective, there is a possibility of being underestimated or overestimated, and therefore, prudent interpretation of results is needed.

## 5 CONCLUSIONS

Within the limitations of this study, we verified that DPBM is biocompatible and can be used as a scaffold to enhance the clinical and radiological outcomes of periodontal regeneration of one‐wall intrabony defects. In particular, the adjunctive use of EMD in combination with DPBM significantly reduced the postoperative discomfort when compared with DPBM without EMD.
